# Localizing Optic Disc in Retinal Image Automatically with Entropy Based Algorithm

**DOI:** 10.1155/2018/2815163

**Published:** 2018-02-06

**Authors:** Lamia AbedNoor Muhammed

**Affiliations:** Computer Sciences and IT College, University of Al-Qadisiyah, Al-Qadisiyah, Iraq

## Abstract

Examining retinal image continuously plays an important role in determining human eye health; with any variation present in this image, it may be resulting from some disease. Therefore, there is a need for computer-aided scanning for retinal image to perform this task automatically and accurately. The fundamental step in this task is identification of the retina elements; optical disk localization is the most important one in this identification. Different optical disc localization algorithms have been suggested, such as an algorithm that would be proposed in this paper. The assumption is based on the fact that optical disc area has rich information, so its entropy value is more significant in this area. The suggested algorithm has recursive steps for testing the entropy of different patches in image; sliding window technique is used to get these patches in a specific way. The results of practical work were obtained using different common data set, which achieved good accuracy in trivial computation time. Finally, this paper consists of four sections: a section for introduction containing the related works, a section for methodology and material, a section for practical work with results, and a section for conclusion.

## 1. Introduction

Glaucoma is a chronic eye disease, which can be controlled but cannot be cured. If left untreated, loss of vision occurs gradually, potentially leading to blindness. The detection and diagnosis of glaucoma are related to tracing the changes in the optic cup which is a portion of optic disc (OD). To perform this detection in an automated system, the optical disc region must be extracted from retinal image through segmentation process. However, localization of optic disc is an important step in simplifying this segmentation. Different methods have been proposed for localization of OD [1]. They have exploited some of OD region features, such as its yellow color, having more brightness, having high grey intensity, and containing a network of convergence vessels. They have been applied through different techniques.

Simple operations were adopted through the works such as Akram et al. [2] who used average filtering and threshold. Aquino et al. [3] exploited morphological operations and edge detection. Whardana and Suciati [4] combined two techniques: morphological operators and clustering with *k*-means method. Li and Chutatape [5] presented an algorithm that uses the clustering of image according to the bright pixel and the candidate regions are passed through principal component analysis (PCA) in order to locate the center of optic disc region. Padmanaban and Kannan [6] suggested the use of Fuzzy C-Means (FCM) clustering. Foracchia et al. [7] worked on tracing the vessels, matching their path with directional pattern in OD in originate image. Nergiz et al. [8] introduced a study using vasculature geometry character in optical disc, convergence to its center. Mendonace et al. [9] presented new methodology: entropy based on the information resulting from the distribution of vessel through the optic disc region.

Learning techniques has good opportunity in optic disc localization fields; however, OD template would be learned based on different features. While Wu et al. [10] worked to build a specific model for network vessels shape in OD region which has form as parabolic shape, Dehghani et al. [11] suggested building a histogram-template for colors which OD region contains. Akyol et al. [12] proposed an algorithm which consists of a multiple-steps algorithm, with induction classifier being one of these steps. The study by Ichim and Popescu [13] used adaptive local texture analysis to generate several features that pass through classification algorithm. Muangnak et al. [14] used in their work decision model, which is induced from direction vectors that were derived from the vessel network, and points of convergence and then used hybrid method. Sinthanayothin et al. [15] applied neural network in their work with data input which is extracted from principle components analysis of the image in question.

Frequency transform techniques have been used in other studies. Pallawala et al. [16] proposed using Daubechies wavelet transform. Jafariani and Tabatabaee [17] employed Fourier-Mellin transform in their work. While another study produced by Esmaeili et al. [18] used curvelete transform technique.

## 2. Materials and Methods

### 2.1. Optic Disc

**Figure 1:**
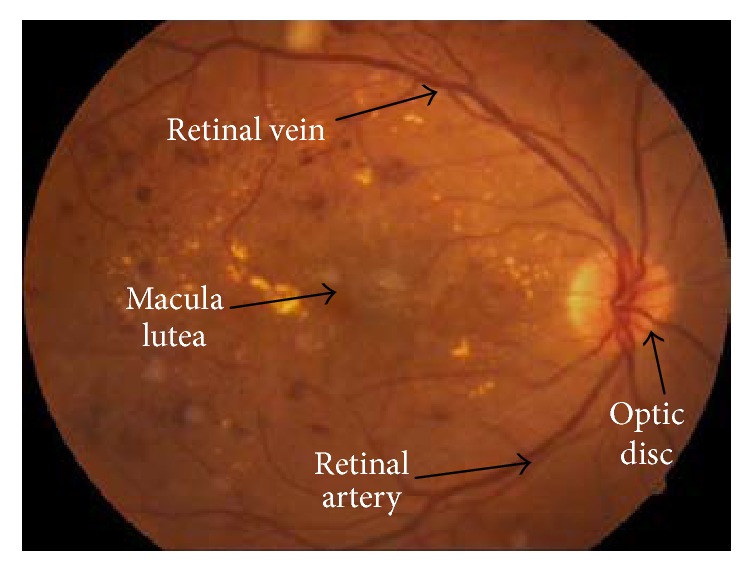
Retinal fundus image [19].

Eye fundus plays an important role in detecting various eye diseases; it consists of retina which is the transparent, light-sensitive structure at the back of the eye, optic nerve disc, and blood vessels (retinal arteries and veins). Anatomically, the retina contains structures including macula which is the central area, rods which are photoreceptor cells that surround the macula, the optic nerve which carries signals, and blood vessels as shown in Figure 1 [19].

One of the problems that eye suffers is the vision loss resulting from the diabetes complications that can be noticed through changes occurring in the retina components. In relation to this, fundus image is used continuously to examine and assess the eye health conditions [2].

Identification of optic disc in fundal landmark is important as reference coordinates to locate anatomical components in retinal images, for vessel tracking as a reference length for measuring distances in retinal images, and for observing any change in the optic disc which may result from a disease [21].

With the development of digital imaging and computing power, the potential to use these technologies in ophthalmology analysis and computer vision techniques also increases. Optic disk localization with this technique has been performed successfully according to its features that can be used in image analysis such as brightness, high contrast, and yellowish disk, where the blood vessels and optic nerves pass through it [2]. Therefore optic disc area contains more detailed information that can be exploited in its identification.

### 2.2. Entropy

An information theory is associated with information measure that has played an important role in different application fields, such as image analysis. The entropy of a probability distribution can be interpreted not only as a measure of uncertainty but also as a measure of information. As a matter of fact, the amount of information acquired from the observation of the result of an experiment (depending on chance) can be taken numerically equal to the amount of uncertainty concerning the outcome of the experiment before carrying it out [22].

Formally, let *X* be a discrete random variable with alphabet *X* and probability mass function *p*(*x*), *x* ∈ *X*. The Shannon* entropy *of *X* is defined as [23]

(1) $$\begin{array}{c} H\left(\;X\;\right)=\Sigma_{x\in X}p\left(\;x\;\right)\log p\left(\;x\;\right), \end{array}$$

where *p*(*x*) ∈ [0.0,1.0],  (−log *p*(*x*)) is an information association for occurrence *x*, and Σ_*x*∈*X*_*p*(*x*) = 1.

### 2.3. Entropy in Image Analysis

A digital image consists of small units that represent brightness of a particular position in the image, called picture elements (pixels). The variation of the pixels values carries information in an image, so it can be measured by entropy metric. However, the image has different distributed brightness values; entropy of image (*I*) can be computed as

(2) $$\begin{array}{c} H_{I}=-\overset{M_{g}-1}{\underset{j=0}{\sum }}p\left(\;j\;\right)\log_{2}p\left(\;j\;\right), \end{array}$$

where *p*(*j*) is the distribution of brightness value indexed by *j* in image *I*; *M*_*g*_ is total of brightness levels in an interested image (*I*).

In image processing, entropy measure generates a value which represents new feature that can be exploited in image analysis such as texture analysis. Low values of entropy refer to smoothing texture, while texture with more details has higher entropy values [24]. Thus, it can be used to generate a new feature to measure the smoothing of the texture of images.

As related, the optic disc area in fundus image contains more details such as nerves and vessels passing through that means its texture is not smooth, so it is expected that its entropy value would be higher than other regions in the fundus image.

### 2.4. Sliding Window Approach

This is an approach that has been used in locating object in an image. However the image is partitioned into subimages (regions); they will be evaluated separately according to quality function. So the region with maximum score will be candidate to be Region of Interest [25].

**Definition 1 .**

Let *X* be an image that would be partitioned into *n* regions (*R*):

*X* = {*R*_1_, *R*_2_,…, *R*_*n*_}.

However, *R*_*i*_ is a region labeled with (top, bottom, left, and right) coordinates.

*f*(*R*_*i*_) is quality function; *xj* is the candidate region with

(3) $$\begin{array}{c} R_{j={\mathrm{argmax}}_{R_{i}\in X}f\left(R_{i}\right)}. \end{array}$$

**Figure 2:**
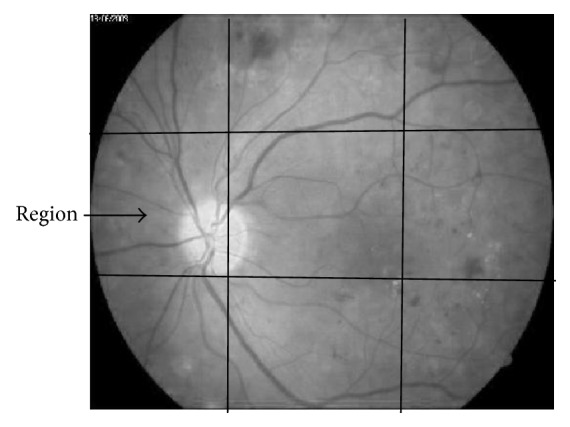
Nonoverlap sliding window.

The proposed algorithm in this paper used sliding window approach with two methods: nonoverlap and overlap. However, in nonoverlap method as shown in Figure 2, each image pixel must not belong to more than one region, so

(4) $$\begin{array}{c} R_{i}\cap R_{j}=\emptyset . \end{array}$$

**Figure 3:**
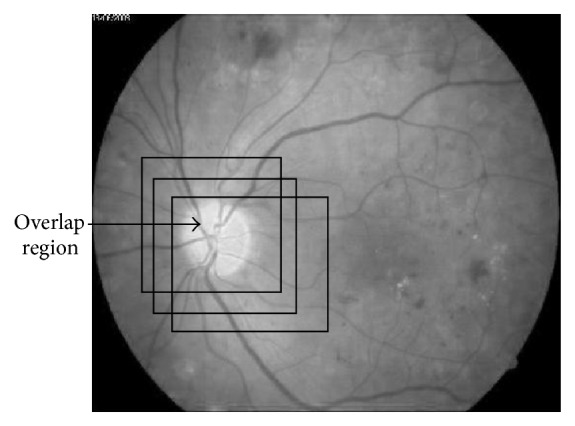
Overlap sliding window.

Meanwhile, overlap method as shown in Figure 3 permits image pixel to belong to more than one region.

(5) $$\begin{array}{c} R_{i}\cap R_{j}\neq \emptyset . \end{array}$$

*R*_*i*_, *R*_*j*_ are regions in the image.

## 3. Practical Work and Results

### 3.1. Proposed Algorithm

As mentioned in the last section, there are some concepts that would be exploited in this work as shown in Algorithm 1.

**Figure 4:**
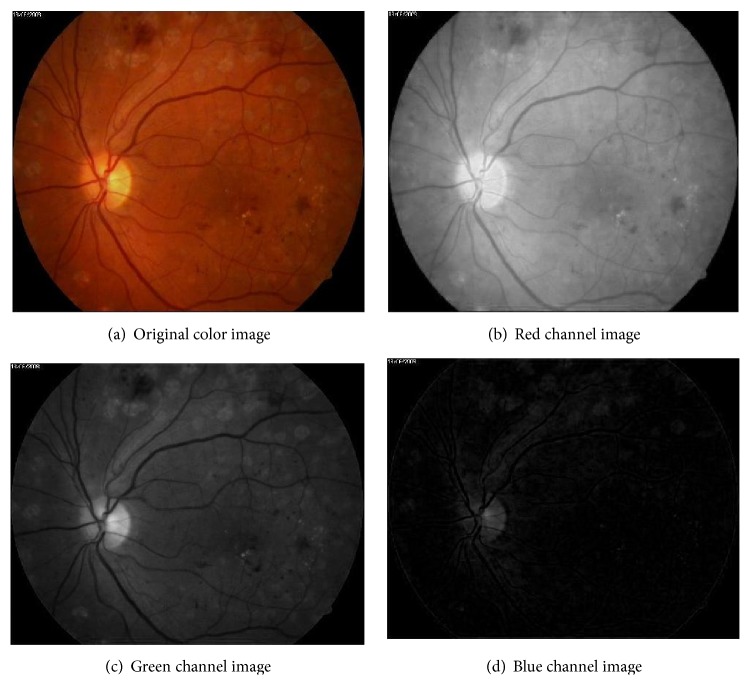
Original image: (a) colors image and (b) red, (c) green, and (d) blue channel image.

1. However, entropy value in the optic disc area is significant; it would be used to find this area. So the algorithm uses searching technique; greedy method was used in this work to find the maximum entropy value.
2. Sliding window technique was used in order to find specific area from the whole image; the image area would be partitioned into patches. This technique was executed in two methods: nonoverlap sliding window in order to find the approximate optic disc area and then using this area with another method; overlap sliding window which is of importance in order to find the location of optic disc accurately.
3. Finally, we used green channel image in this work according to results from previous related experimental work about this image. It seems from subjective information about image channels (red, green, and blue) that the best one is green channel image; however it gives maximum contrast [26]. Therefore its texture is more distinguished than other channel images as shown in Figure 4.

### 3.2. Experimental Work

The proposed algorithm was executed computationally using the following tools: MATLAB R2014a programming language and computer with specification Intel®Core™i5-3320M CPU@2.60 GHz; tests were applied with the known data sets: DRIVE, CHASEDB, DRIONS-DB, and DIARETDBI. In addition, a special data set is used [20], whose results will be given in detail in the following paragraphs.

**Figure 5:**
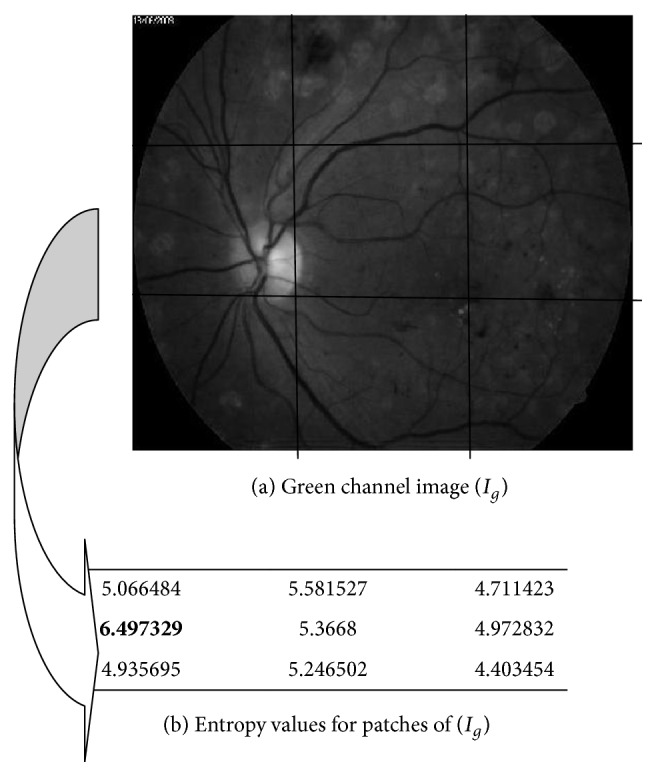
Image *I*_*g*_ number (29) in data set with its nonoverlap patches entropies.

The algorithm was initiated with extraction of green channel image (*I*_*g*_) which was extracted from original image (*I*) as shown in Figure 4. So image (*I*_*g*_) was divided into (3 × 3) patches (*R*) using nonoverlap sliding window as shown in Figure 5(a). The size of each patch is the same; it is approximate to size of optic disc area. From the experimental work here, this size is suitable for implying more of optic disc area in one of the patches, so significant patch that may contain optic disc comes out. Then, for each patch *R*_*i*,*j*_, entropy value would be generated as shown in Figure 5(b).

**Figure 6:**
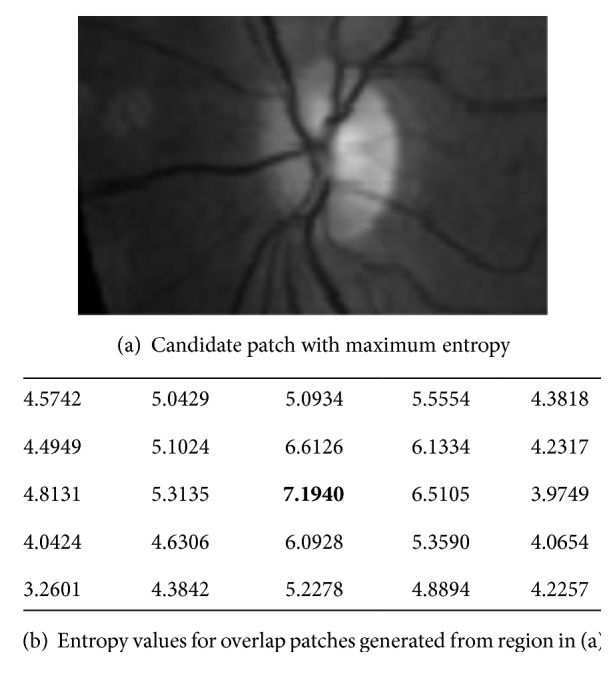
New region (*R*_new_) with its overlap patches entropies.

**Figure 7:**
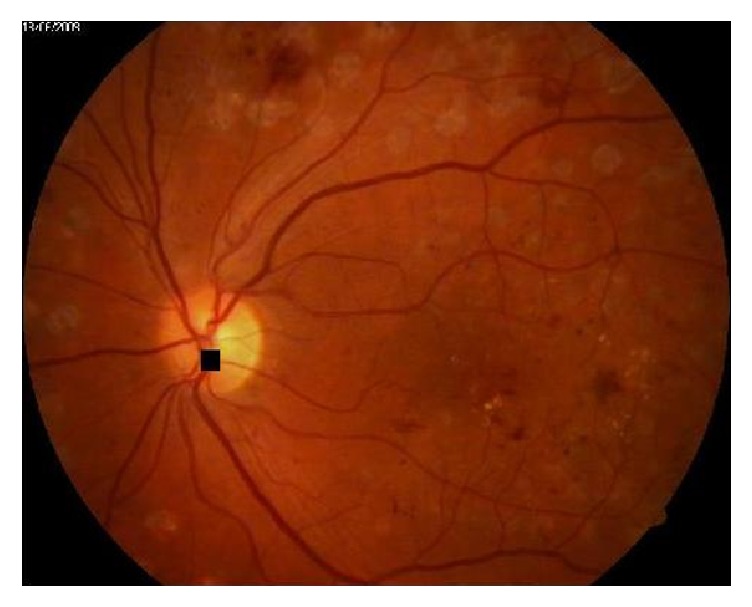
Original image with marking optic disc location.

The patches are ranked according to their entropy values. The one with the highest entropy value would be selected as the area that may contain optic disc, as shown in Figure 5(b). The darker box referring to the highest value which is corresponding to the patch is addressed by *R*_2,1_ as shown in Figure 6(a). New region (*R*_new_) would be generated from patch *R*_2,1_, and half of each surrounded patch of *R*_2,1_. However, the existence of these halves in a new patch guarantees the possibility that some optic disc area may be in one of the surrounded patches. Then *R*_new_ would be divided into overlap patches as (4 × 4), (5 × 5), or (6 × 6). From the experimental work, this number of divisions is suitable; however, size of new patches is approximately equal to the size of optic disc area as shown in Figure 6(b). The new patches entropies would be measured. The patch with maximum value would be candidate to be the optic disc area. From the results shown in Figure 6(b), the patch that is indexed (*R*_new_3,3__) has the highest entropy value. Finally, small black square is placed in the center location of this patch as shown in Figure 7.

### 3.3. Results and Findings

**Figure 8:**
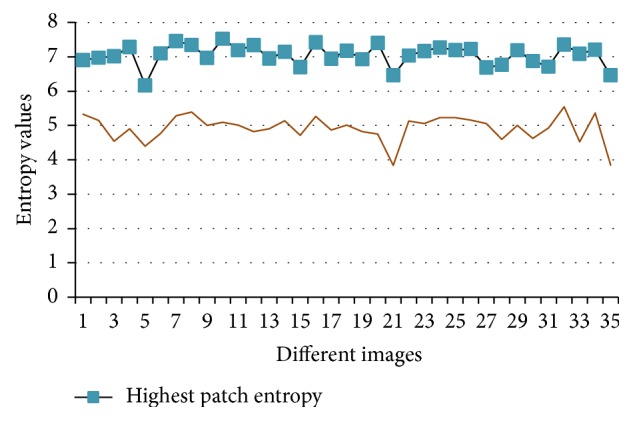
Comparison of the entropy value of candidate patch versus the entropy mean of all patches in each of the 35 tested images from source [20].

**Figure 9:**
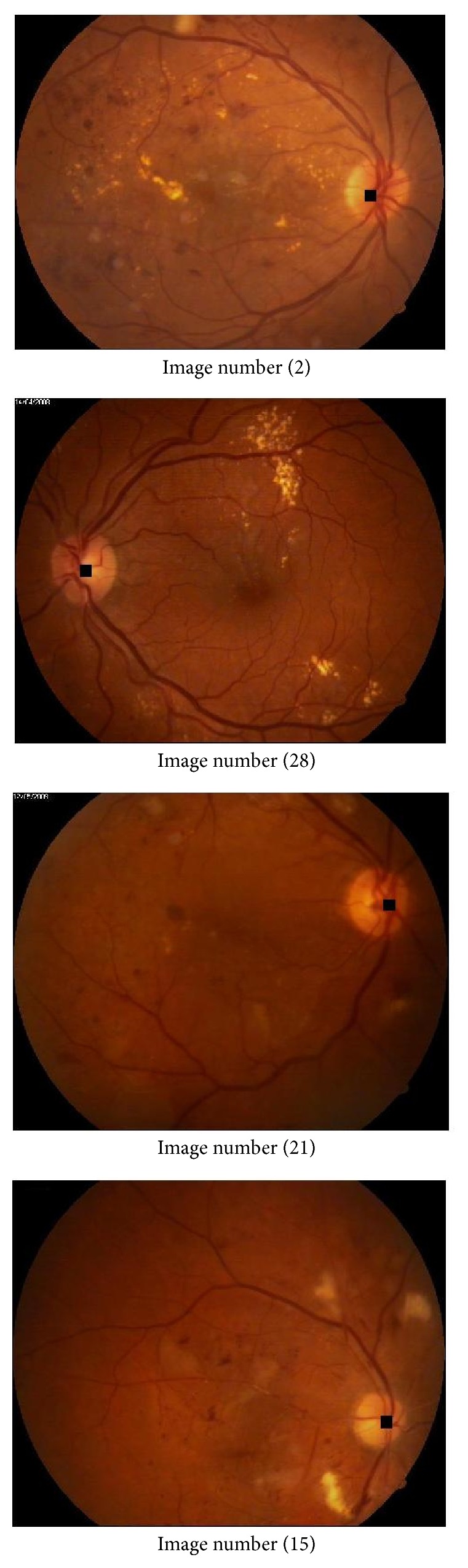
Sample output marked images.

As related to data set [20] results, the computing entropies for candidate patch that is closer to optic disk area are more significant than other patches in image; this is true for all the images (35 images) as shown in Figure 8. However there is a comparison between candidate patch entropy value and the mean entropy values of the rest of the patches in an image; each one is represented by different line. It is obvious that candidate patch entropy value exceeds the mean patches entropies, for all tested images. So these images would be marked correctly. Sample of these marked images is shown in Figure 9. In addition, the experimental work would be applied with other data set and two measures were computed for comparison the results: accuracy of localization of OD in each data set image and execution time as shown in Table 1. There is a disparity in the accuracies values of OD localization, but the time execution is stable for different tested data because the executed steps are constant.

## 4. Conclusion

Researchers have focused on localization of optic disk using a computer, so more methods have been suggested with good results. In spite of this, the proposed algorithm in this paper gives significant results through simple computational steps that are executed in a short space of time; however there is no need for any preprocessing enhancement steps. Moreover, the proposed algorithm is attractive; it entails a simple technique, so it can be combined with other algorithms in order to be more effective as a supporting step.

Finally, there are more features that are significant in the OD area, so in future work combining them with the entropy feature in order to generate robust algorithm is suggested. Also the evolutionary algorithm can enhance this algorithm to give accurate localization.

## Figures and Tables

**Algorithm 1:**
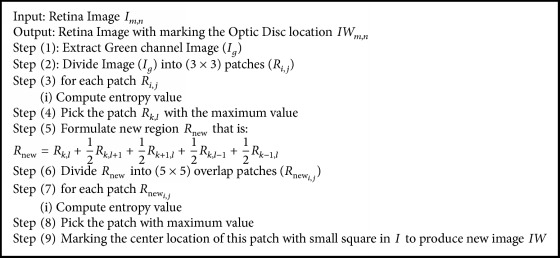
Proposed algorithm: localization of optic disk.

**Table 1:** Comparison of the results for different data sets.

Data set name	Measure		
	Number of images	Accuracy	Average time of execution (millisecond)
CHASEDB (*R* + *L*)	28	75%	37.641
DRIONS-DB	110	77%	44.305
DRIVE	40	95%	36.9
DIARETDBI	89	89%	38.12
Special data set [20]	35	100%	37.955
